# UV random laser emission from flexible ZnO-Ag-enriched electrospun cellulose acetate fiber matrix

**DOI:** 10.1038/s41598-019-48056-w

**Published:** 2019-08-13

**Authors:** Manoel L. da Silva-Neto, Mário C. A. de Oliveira, Christian T. Dominguez, Raquel E. M. Lins, Nikifor Rakov, Cid B. de Araújo, Leonardo de Souza Menezes, Helinando P. de Oliveira, Anderson S. L. Gomes

**Affiliations:** 1 Programa de Pós-Graduação em Ciências de Materiais, Universidade Federal de Pernambuco, Recife, 50670-901 PE Brazil; 2 Pós-Graduação em Ciência dos Materiais, Universidade Federal do Vale do São Francisco, 48902-300 Juazeiro, BA Brazil; 3 Departamento de Física/CCEN, Universidade Federal da Paraíba, João Pessoa, 58051-900 PB Brazil; 4 Departamento de Física, Universidade Federal de Pernambuco, Recife, 50670-901 PE Brazil

**Keywords:** Electronic properties and materials, Polymers

## Abstract

We report an alternative random laser (RL) architecture based on a flexible and ZnO-enriched cellulose acetate (CA) fiber matrix prepared by electrospinning. The electrospun fibers, mechanically reinforced by polyethylene oxide and impregnated with zinc oxide powder, were applied as an adsorbent surface to incorporate plasmonic centers (silver nanoprisms). The resulting structures – prepared in the absence (CA-ZnO) and in the presence of silver nanoparticles (CA-ZnO-Ag) - were developed to support light excitation, guiding and scattering prototypes of a RL. Both materials were excited by a pulsed (5 Hz, 5 ns) source at 355 nm and their fluorescence emission monitored at 387 nm. The results suggest that the addition of silver nanoprisms to the ZnO- enriched fiber matrix allows large improvement of the RL performance due to the plasmon resonance of the silver nanoprisms, with ~80% reduction in threshold energy. Besides the intensity and spectral analysis, the RL characterization included its spectral and intensity angular dependences. Bending the flexible RL did not affect the spectral characteristics of the device. No degradation was observed in the random laser emission for more than 10,000 shots of the pump laser.

## Introduction

Recently, burgeoning growth of attention has been devoted to the development of flexible photonic systems^1^ due to their potential applications as light emitting devices, foldable displays and wearable sensors^2,3^. Organic and inorganic materials have been exploited for displays or light emitting devices^4,5^. Among the optical sources of interest described here, flexible random lasers (RLs) have been proposed for such applications^6–11^. As reviewed by Feng *et al*.^12^ and Wiersma^13^, RLs are optical sources which are characterized by their unique lasing mechanism: feedback due to light scattering. While in conventional lasers the feedback is provided by static reflecting mirrors, in RLs it is provided by random scatterers, as first proposed by Letokhov^14^. It is now well accepted that RLs have opened a new perspective for partially coherent light sources^15–17^. Among the materials exploited as gain medium for RLs, ZnO nanostructures stand as a versatile alternative, which can be optically^18,19^ or electrically pumped^20,21^, and incorporated in a diversity of hosts^22,23^, besides the fact that it can itself be the scattering medium^18^. In particular, ZnO RLs have shown high efficiency under single and multiphoton excitation^18,24,25^ allowing a wide range of excitation wavelengths. Furthermore, ZnO RLs could hold important applications from high-resolution bioimaging, multiphoton microscopy, laser therapy and optical storage^26–29^.

Random lasing action arising from electrospun nanofibers, which act as the scatterers, doped with appropriate gain medium, has been recently studied as reported in refs^30–34^ introducing a new class of flexible RLs. Cellulose – the most reported and common biopolymer in the world and the widely used in the ester form (in the present case cellulose acetate - CA)^35,36^ are important and relevant building blocks with a plenty of green credentials for different applications. Moreover, the use of devices in nanoscale regime^37^ offers a diversity of new applications for these nanostructured materials. In particular, the production of electrospun fibers of CA introduces advantages for biomedical materials due to their superior chemical resistance, biocompatibility and biodegradability^38^. On the other hand, the cylindrical structure offers an adequate condition for light propagation and scattering, being possible the development of organic RL structures^30^.

In the current study, we fabricated and exploited the use of electrospun fibers of cellulose acetate modified with ZnO and decorated with silver nanoparticles^39^ to produce stable UV RL emission.

## Results

### Structural and morphological characterization

**Figure 1:**
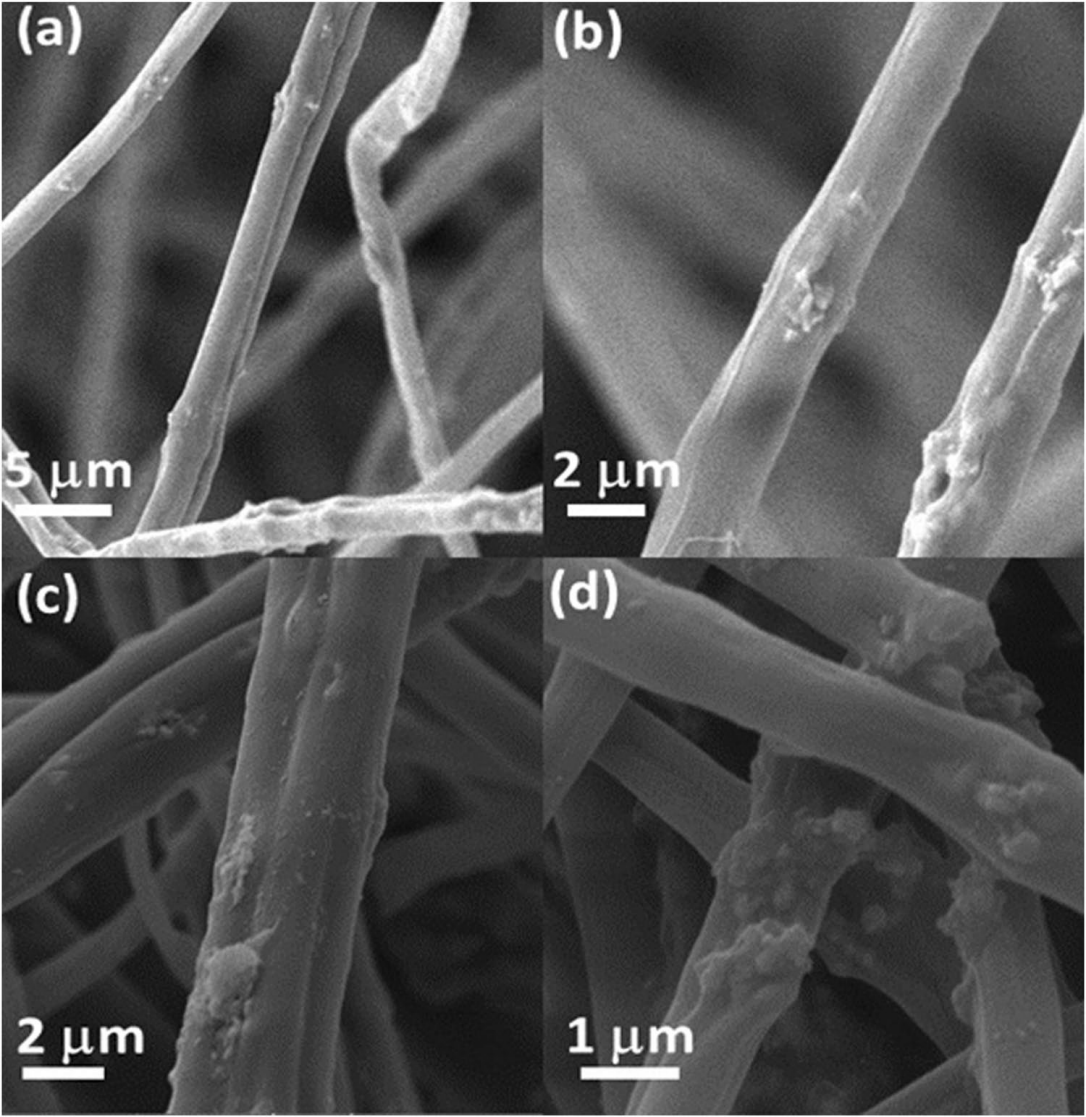
SEM images of electrospun CA fibers (**a**) and (**b**) with CA-ZnO fibers, (**c**,**d**) with CA-ZnO-Ag fibers.

The morphology and structure of electrospun fibers were evaluated by scanning electron microscopy (SEM) and Fourier-Transform Infrared Spectroscopy (FTIR) spectra. SEM images, shown in Fig. 1a,b (sample: CA-ZnO), confirm that resulting fibers (prepared in the absence/ presence of additives – ZnO and Ag nanoparticles) are regular structures and free of beads. The resulting fibers of CA-ZnO present average diameter of (1.79 ± 0.61) µm. Due to the process of silver nanoparticles adsorption, the diameter of the resulting fibers increases to a value of (2.53 ± 1.08) µm (see Fig. 1c,d) with surface morphology modification in response to the silver nanoparticles deposition. It is a consequence of slight swelling of fibers immersed in silver nanoparticles suspension for adsorption of silver nanoparticles on the outer surface of the fibers.

**Figure 2:**
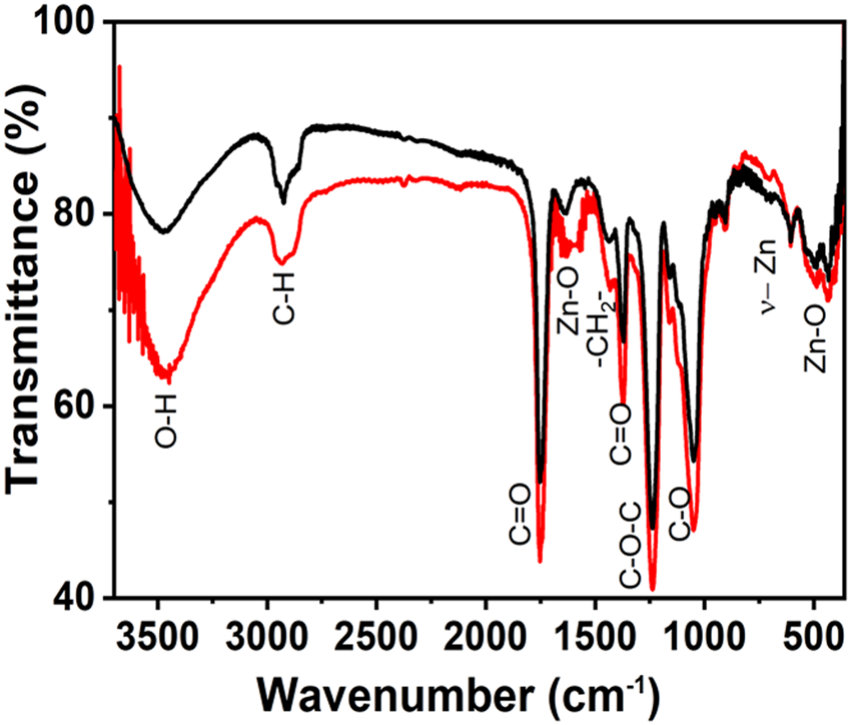
FTIR spectra of samples CA-ZnO (black line) and CA-ZnO-Ag (red line). The numbers indicate the corresponding assignments for the characteristic vibration energies (see text for details).

**Figure 3:**
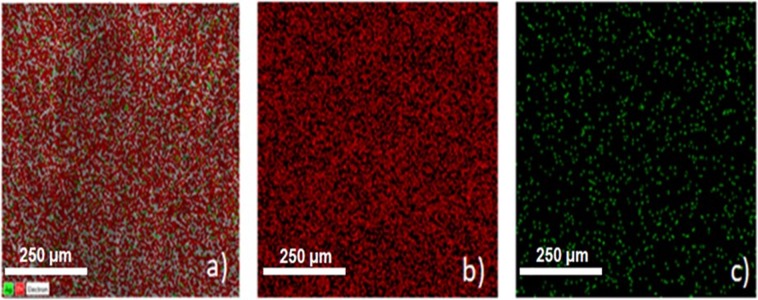
Overlaid EDS images for sample containing CA-ZnO-Ag (**a**) with corresponding identification of components: zinc in red (**b**) and silver in green (**c**).

In terms of structure, a scrutiny of the FTIR from 3700 cm^−1^ to 400 cm^−1^ was performed to evaluate the composition and possible interaction of components in electrospun fibers. For comparison, FTIR spectra of samples prepared in the absence and in the presence of adsorbed silver nanoparticles are shown in Fig. 2. Characteristic peaks are observed at 3471 cm^−1^ (stretching of intermolecular OH and hydroxyl groups)^40–42^, 2928 cm^−1^ (C-H stretching vibration)^40^, 1752 cm^−1^ (carbonyl group (C=O) stretching vibration – characteristic of pure cellulose acetate)^42^, 1633 cm^−1^ (Zn-O stretching vibration)^41^, 1431 cm^−1^ (–CH_2_- deformation vibration for pure CA)^43^, 1374 cm^−1^ (C=O asymmetric vibration^40^, 1237 cm^−1^ (‘C-O-C’ anti symmetric stretching vibrations of ester group -CA)^43^, 1051 cm^−1^ (C-O stretching vibration), 899 cm^−1^ (formation of tetrahedral coordination of Zn), 709 to 603 cm^−1^ due to the stretching vibrations of ZnO nanoparticles^40^ and 427 cm^−1^ in response of Zn–O stretching vibration^42,44^. The typical superposition of peaks associated to samples prepared in the presence and absence of silver nanoparticles confirms the very close proximity of peaks of silver and zinc acetate, reported in the literature^44^. The distribution of components on electrospun fibers of CA-ZnO-Ag was determined by Energy Dispersive X-Ray Spectroscopy (EDS) images (shown in Fig. 3). As can be seen, a uniform distribution of zinc (red dots) and silver (green dots) is established on the fibers’ surface, characterizing abundant distribution of components on electrospun fibers.

### Random laser behavior

**Figure 4:**
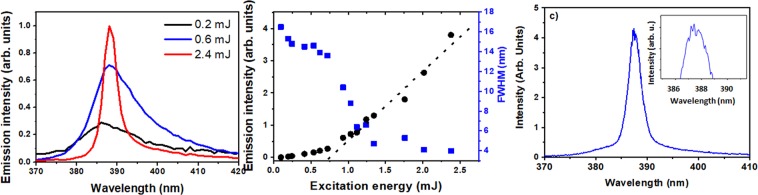
(**a**) Averaged spectral emission from below (black dashed line –excitation energy 0.2 mJ – and red long dashed line, excitation energy 0.6 mJ) to above (blue continuous line– excitation energy 2.4 mJ) threshold for sample CA-ZnO-Ag; (**b**) Emission spectral linewidth (blue squares) and emission intensity (black circles) dependence on excitation energy at 5 Hz. The dashed straight line indicates the threshold energy on the horizontal scale. (**c**) a single shot spectrum (excitation above threshold), in which spikes (see inset in **c**) can be seen.

We first characterized the CA-ZnO samples. Shown in Fig. 4(a–c) are the spectral and intensity dependence of the RL as a function of excitation pulse energy. The inset in Fig. 4c is a zoom around the spectral RL emission obtained with a single shot measurement, instead of averaging (as in 4a). It shows the presence of spikes, characteristic of the modes of the RL (which are otherwise averaged out).

**Figure 5:**
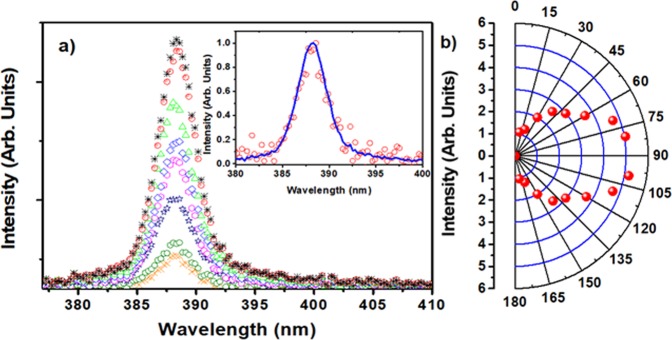
(**a**) Spectra of RL emission for different observation angles between 10° (black line, small peak) and 80° (brown line, large peak) with 10° intervals with respect to the sample surface. The inset shows the normalized RL intensity at 10° (red open circles) and 80° (blue line); (**b**) angular dependence of the RL peak intensity in the range 10°–170°. Sample: CA-ZnO-Ag.

As depicted in Fig. 5(a), we studied the angular dependence of the RL emission for excitation above threshold (pump pulse energy: 2.4 mJ). The inset of Fig. 5(a) shows that the spectral width for the RL emissions detected at 10° (red open circles) and 80° (solid blue line) is the same. This is, in fact, observed for all spectra detected at various angles from 10° to 170° (except for the position at 90°, not measured due to the way the setup is conceived), therefore confirming that ASE contribution is minimum (ASE is higher at the edge direction due to waveguide behavior of light into the sample) and RL emission is occurring.

Although in the majority of reports on random lasing the authors do not discuss the RL degradation in relation to the excitation pulse energy and/or the excitation laser repetition rate, this effect is particularly detrimental in RL with dyes as the gain medium^45^. In the present case, no degradation was observed up to 4 × 10^4^ shots incident upon the sample for an excitation pulse energy of 2.5 mJ, well above threshold (the RL peak intensity was constant within 0.4%). This is a much better performance than the reported degradation behavior in dye-based RLs^45,46^, where it already degraded after less than 200 shots, unless the nanoparticles are modified as shown by Pincheira *et al*.^45^.

**Figure 6:**
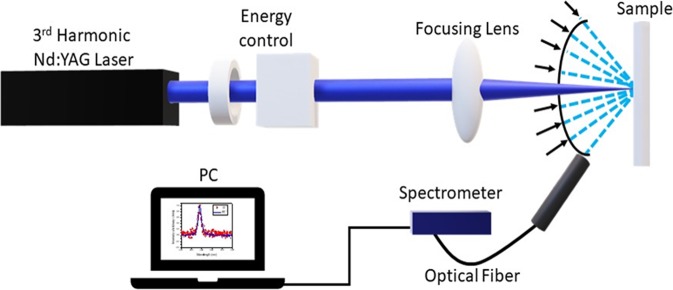
Experimental scheme for ZnO-based optical characterization. The 3rd harmonic of Nd:YAG laser passed through a half-wave plate and a polarizer for energy control. Then it was focused by a 50 cm focal length lens onto the sample surface. The angular distribution of RL emission was measured by changing the optical fiber position as represented by the various arrows close to the sample in the scheme.

**Figure 7:**
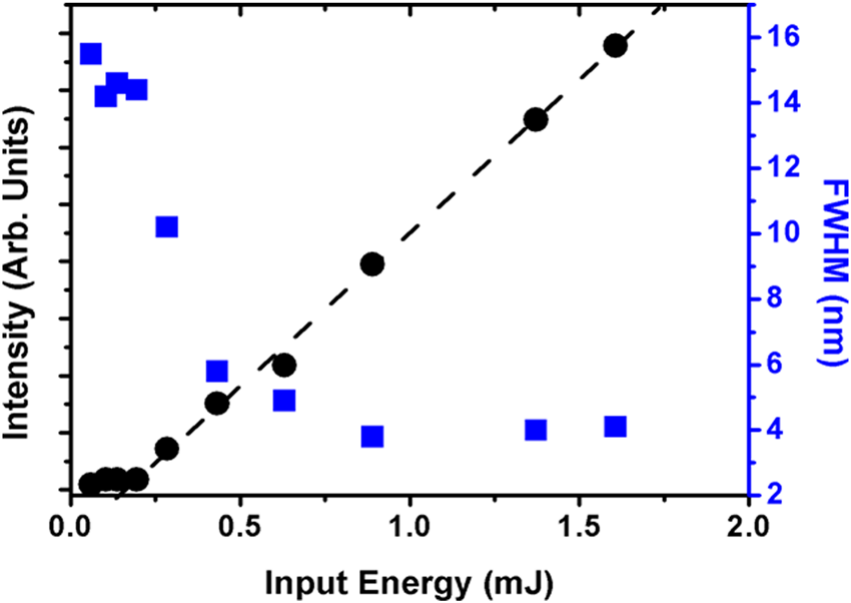
Emitted intensity (black circles) and emission bandwidth (blue squares) in the CA-ZnO-Ag RL – a clear reduction of the RL threshold due to the presence of the Ag NPs can be observed (compare with Fig. 4b).

The CA-ZnO-Ag RL sample was evaluated using the same apparatus shown in Fig. 6 and the results are shown in Fig. 7 for 50 nm Ag nanoprisms incorporated in the flexible RL matrix.

Figure 7 should be directly compared to Fig. 4(b), as they were obtained under the same excitation conditions. While the bandwidth narrowing was similar in both cases, the threshold is clearly reduced from ~0.74 mJ to ~0.15 mJ with the incorporation of the 50 nm Ag nanoprisms.

**Figure 8:**
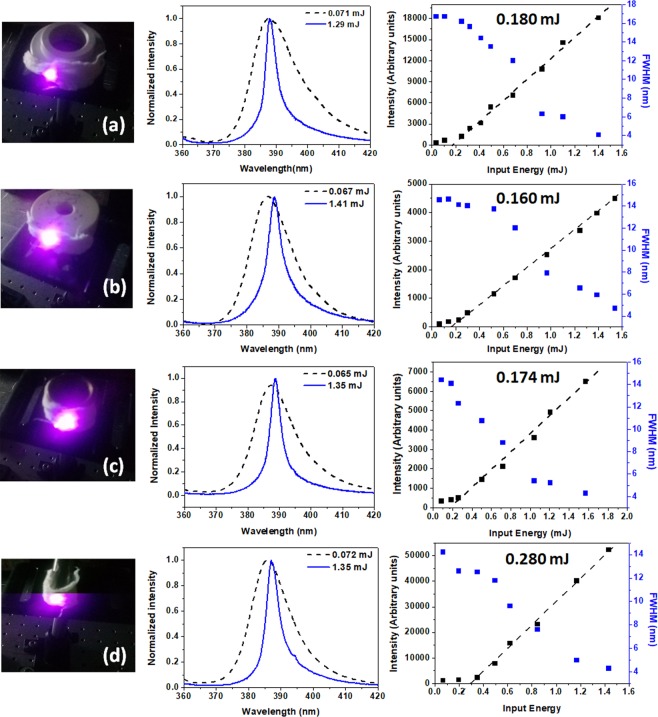
Normalized intensity vs. input energy and corresponding variation in intensity/FWHM measured at different bending radius of electrospun fibers (curvature of 0.22 cm^−1^ (**a**), 0.25 cm^−1^ (**b**), 0.40 cm^−1^ (**c**) and 0.83 cm^−1^ (**d**)). The pictures show the excitation laser position on the sample.

To evaluate the flexibility degree of the electrospun fibers, we performed a set of experiments in which the RL properties of fibers were characterized varying the bending radius. As summarized in Fig. 8, the measured threshold energy for RL emission presents reduced variation with decreasing the bending radius of the electrospun matrix (curvature from 0.22 cm^−1^ to 0.83 cm^−1^), with the peak emission wavelength also constant around the maximum non-bent emission of 387 nm, confirming that the optical properties of the resulting matrix are preserved at different curvatures, potentializing important applications for the flexible RL device. Note that at severe bending condition, it is observed an increase in the threshold energy (~0.280 mJ) as a consequence of progressive decrease in the excitation intensity at the borders of the excited area in comparison with the center of the sample.

## Discussion and Conclusions

ZnO nanoparticles incorporated in cellulose acetate fiber matrix were prepared by electrospinning, and the system was characterized as a RL pumped by nanosecond laser pulses. This novel RL architecture had its performance comparable to other flexible fiber based RL, most of them using dyes as the gain medium^30–34^. The incorporation of silver nanoprisms to the RL matrix gave rise to a plasmonic effect in the RL emission, which led to an ~80% reduction in the threshold energy due to an enhanced electric field around the metallic nanoprisms/dielectric medium interface, from which the ZnO emission directly benefited. It is worth mentioning that, using Ag nanoprisms of size larger than 150 nm no plasmonic effect was observed, as expected from the relative size of such nanoparticles (in agreement with previous results reported in the literature^47,48^ about the influence of plasmon effects on reduction of the lasing threshold). One important aspect of using ZnO as the gain medium, in comparison with organic dyes, is the fact that there is no observable degradation effect from the interaction of the pump with the gain medium, very common in dyes based RLs^30,45^. It should be noticed that ref.^30^ uses as the scattering material a similar nanofiber system as used here but a dye was the active laser medium. In that case, the RL intensity decreased to 74% of the maximum intensity after 2200 shots at 1 Hz using similar pump conditions. The present results showed a stable RL emission intensity even after 3.5 × 10^4^ shots (~120 min), with no indication that this would change in the long (few hours) term. Another characterization in our system was the RL intensity dependence on observation angle. Results as the study performed by Wu *et al*.^49^, in flat samples, shown theoretically and experimentally that the increment of scatterers concentration modifies the shape of excited region, from a cone at relative lower concentrations to a hemisphere at relative higher concentrations. Similar studies performed later by other groups in cylindrical samples^50,51^, showed that the angular distribution of far-field intensity of RL is oval in shape. Figure 5 showed that the distribution of the far-field intensity of our RL is oval too, being more intense in the direction perpendicular to the sample surface than in the edge direction.

From results in Fig. 5(a,b), we infer that RL feedback happened mainly in the backscattering direction intensifying the RL emission in the perpendicular direction, and this preferential feedback and the light attenuation limits the RL intensity at edge directions of sample. Nevertheless, the NPs concentration is sufficient to sustain the RL emission, such as the FWHM remains constant at ~4 nm in the range of 10–170^o^, as shown in the inset of Fig. 5(a).

In conclusion, the ZnO based RL reported here demonstrated its robustness which is quite useful for carrying out experiments using this kind of RL as the photon source, for instance for imaging purposes^16^ or for intensity fluctuations statistics studies, which is an interesting characteristic of random lasers^45^. We anticipate that similar devices using rare earth doped nanoparticles, such as trivalent neodymium^52^ or erbium^53^, in the same kind of matrix, can operate in the near infrared (1000 nm to 1500 nm), therefore extending the operating wavelength range to regions of biological interest.

## Materials and Methods

### Materials

Acetyl cellulose, silver nitrate (AgNO_3_), trisodium citrate (TSC), sodium borohydrate (NaBH_4_), poly (ethylene oxide) and zinc oxide (ZnO) (Sigma Aldrich), dichloromethane - DCM (Vetec), ethanolic alcohol and methanol (MeOH) (Vetec) were used as received for the sample’s preparation. All solutions were prepared using deionized water (18 MΩ).

### Preparation of silver nanoprisms

**Figure 9:**
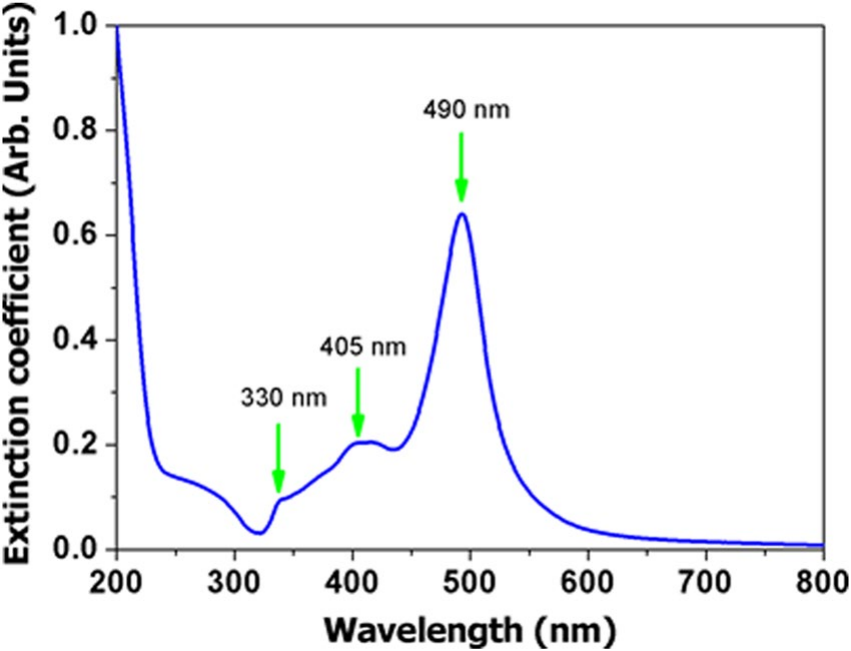
Extinction spectrum of silver nanoprisms in aqueous solution. Notice that the plasmon resonance of the Ag nanoprisms is in resonance with both excitation laser and RL emission wavelengths.

The preparation of silver nanoprisms followed the procedure described in detail by Saade *et al*.^39^ In short, a seed colloid with silver nanospheres (3 to 5 nm diameter) was prepared. 10 µL of NaBH_4_ (20 mM) at room temperature were added into a 30 mL aqueous solution of AgNO_3_ (0.25 mM) and TSC (1 mM) and vigorously stirred. Care was taken not to expose to the environment illumination. The yellow solution was inserted into five 5 mL vials and each vial was irradiated by five arrangements of several LEDs, electrically connected in parallel, with different central wavelength emissions from 467 nm to 630 nm. In the present case, 475 nm was chosen as the preferred wavelength (see ref.^39^ for further details). The extinction spectrum of the colloidal solution of Ag nanoprisms in TCS, similar to that of Fig. 3 in ref.^39^, is shown in Fig. 9, which is characteristic of the prepared nanoprisms, showing the peaks at 330 nm, 405 nm and 490 nm. The spectrum was obtained with a digital PerkinElmer Lambda 650 spectrophotometer.

### Electrospinning procedure for CA-ZnO production

The polymeric solution for electrospinning was prepared as follows: acetyl cellulose (10 wt%) was dispersed in a 5 mL-solution of DCM/ MeOH (4:1 v/v) and kept under intense stirring. Zinc oxide (40 wt% of CA) was slowly incorporated into the polymeric solution under continuous stirring (2 min) to avoid formation of aggregates. After that, poly (ethylene oxide) (PEO - 10 wt % of CA) was spilled into the mixture and dispersed under stirring for additional 3 min. The freshly prepared solution was introduced in a 5 mL syringe and kept under constant pressure in an infusion pump (infusion rate of 1.5 mL/h) for use in the electrospinning assays. The dip of the spinneret was connected to a voltage source of 15 kV that was kept at a fixed distance of 10 cm from the grounded target. The humidity during the deposition process varied in the range of 40–50%. The prepared samples (CA-ZnO) were separated from metallic target and kept under dry environment – reduced humidity.

### Preparation of CA-ZnO-Ag

Electrospun fibers of CA-ZnO (10 mg) were separated for adsorption of 1 mL of silver nanoprisms solution. The adsorption process takes place at 25 °C at humidity of 50%. After complete evaporation of solvent, the sample was kept at dry condition for posterior characterization.

### Random laser characterization

The lasing emission spectra of the studied samples were characterized upon excitation using the third harmonic of a pulsed Nd:YAG laser with a wavelength centered at λ = 355 nm (VIBRANT 355 LD, 5 Hz, 5 ns), delivering a maximum energy of 27 mJ. The excitation beam was perpendicularly directed onto the sample, and gently focused to a diameter of ~2.5 mm at the sample surface. The emitted radiation was collected at ~45° by a multimode fiber placed at a fixed distance of 15 cm from the center of the sample to take the RL emission to an Ocean Optics spectrometer (USB HR4000, optical resolution ~1 nm) for spectral analysis. The RL results were recorded under identical experimental conditions and all measurements were carried out at room temperature. The experimental scheme used is displayed in Fig. 6. For the curvature effect characterization, chosen supports with known radius of curvature were employed to hold the flexible RL, as seen in the pictures of Fig. 8.

## Data Availability

The authors declare that all of data and associate protocols are promptly available to readers.
